# Damage of the Bone Marrow Stromal Precursors in Patients with Acute Leukemia at the Onset of the Disease and During Treatment

**DOI:** 10.3390/ijms252413285

**Published:** 2024-12-11

**Authors:** Aleksandra Sadovskaya, Nataliya Petinati, Irina Shipounova, Nina Drize, Igor Smirnov, Olga Pobeguts, Georgiy Arapidi, Maria Lagarkova, Luiza Karaseva, Olga Pokrovskaya, Larisa Kuzmina, Anastasia Vasilieva, Olga Aleshina, Elena Parovichnikova

**Affiliations:** 1 National Medical Research Center for Hematology, Moscow 125167, Russialoel@mail.ru (N.P.); parovichnikova.e@blood.ru (E.P.); 2 Federal State Budget Educational Institution of Higher Education, M.V. Lomonosov Moscow State University, Moscow 119991, Russia; 3 Lopukhin Federal Research and Clinical Center of Physical-Chemical Medicine of Federal Medical Biological Agency, Moscow 119435, Russia; 4 Shemyakin-Ovchinnikov Institute of Bioorganic Chemistry of the Russian Academy of Sciences, Miklukho-Maklaya 16/10, Moscow 117997, Russia

**Keywords:** multipotent mesenchymal stromal cells, acute leukemia, gene expression, proteome, secretome

## Abstract

In patients with acute leukemia (AL), malignant cells and therapy modify the properties of multipotent mesenchymal stromal cells (MSCs) and their descendants, reducing their ability to maintain normal hematopoiesis. The aim of this work was to elucidate the alterations in MSCs at the onset and after therapy in patients with AL. The study included MSCs obtained from the bone marrow of 78 AL patients (42 AML and 36 ALL) and healthy donors. MSC growth characteristics, gene expression pattern, proteome and secretome were studied using appropriate methods. The concentration of MSCs in the bone marrow, proliferative potential, the expression of several genes, proteomes and secretomes were altered in AL-MSCs. Stromal progenitors had been affected differently in ALL and AML patients. In remission, MSC functions remain impaired despite the absence of tumor cells and the maintenance of benign hematopoietic cells. AL causes crucial and, to a large extent, irreversible changes in bone marrow MSCs.

## 1. Introduction

Multipotent mesenchymal stromal cells (MSCs) are involved in the regulation of hematopoiesis in several ways. They give rise to various stromal cell types and remodel the bone marrow matrix They communicate with hematopoietic stem and precursor cells (HSCs) via paracrine factors and extracellular vesicle secretion [1]. MSCs and their progeny form niches for HSCs in homeostasis and disease [2].

In hematologic malignancies, the stromal microenvironment, including MSCs, is altered under the influence of the developing hemoblastosis and received therapy [3,4]. MSCs can actively and passively regulate the progression of acute leukemia (AL) through cell-to-cell contact, cytokine–receptor interaction and exosome communication [5]. A growing body of evidence suggests the importance of niche alteration in AL progression [6].

The proteome reflects alterations in intracellular processes, and the secretome can give information about the factors affecting normal hematopoietic and leukemic cells. In order to explore the properties of the MSCs of acute leukemia patients (AL-MSCs), we studied their proteomes, secretomes and expression patterns of several genes important for differentiation and hematopoiesis maintenance. We did not separate the secretomes into fractions of paracrine factors and vesicles. We compared AL-MSCs from the patients at the onset of AL to donor MSCs (D-MSCs), AL-MSCs from the patients in remission to D-MSCs and AL-MSCs at the onset to AL-MSCs in remission. The alterations arising at the onset of AL are most likely promoting disease development or caused by malignant cells [7]; the alterations found in remission can be caused by therapy or persist since onset, and among those can be the ones saving residual leukemic cells and allowing AL relapse.

The obtained data suggest dramatic changes in the proteome and secretome of MSCs in patients with acute myeloid leukemia (AML-MSCs) and acute lymphoblastic leukemia (ALL-MSCs), both at the onset of and in remission from AL.

## 2. Results

### 2.1. Comparison of AL-MSC and D-MSC Concentration in the Bone Marrow, Proliferation Potential and Gene Expression Pattern

The aim of this work was to analyze the differences in MSCs obtained from the same patients at the onset of and in remission from AL. MSCs were isolated from bone marrow by the standard method of adhesion to the bottom of plastic flasks, described in more detail in Section 4.2. It was not possible to obtain MSC samples in remission for every patient, which led to the difference in the number of samples.

**Figure 1:**
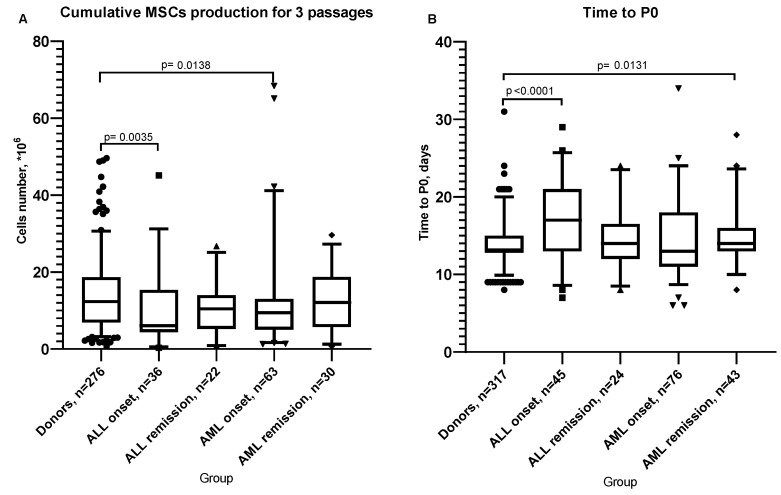
Characteristics of MSC growth. (**A**) Cumulative MSC production for 3 passages. (**B**) Time to Passage 0. Box plots’ whiskers cover 95th percentile. Statistical analysis in all panels was performed using Mann–Whitney test.

Under the same culture conditions, the growth characteristics of D-MSCs and AL-MSCs differed. At the onset of the disease, ALL-MSCs production was significantly lower than that of D-MSCs, and the time to confluency after seeding (time to P0) was significantly increased. The same trend was observed in AML-MSCs. In remission, the growth parameters were restored in ALL-MSCs but not in AML-MSCs (Figure 1).

No significant differences in the analyzed growth parameters were found between MSCs from ALL and AML patients.

**Figure 2:**
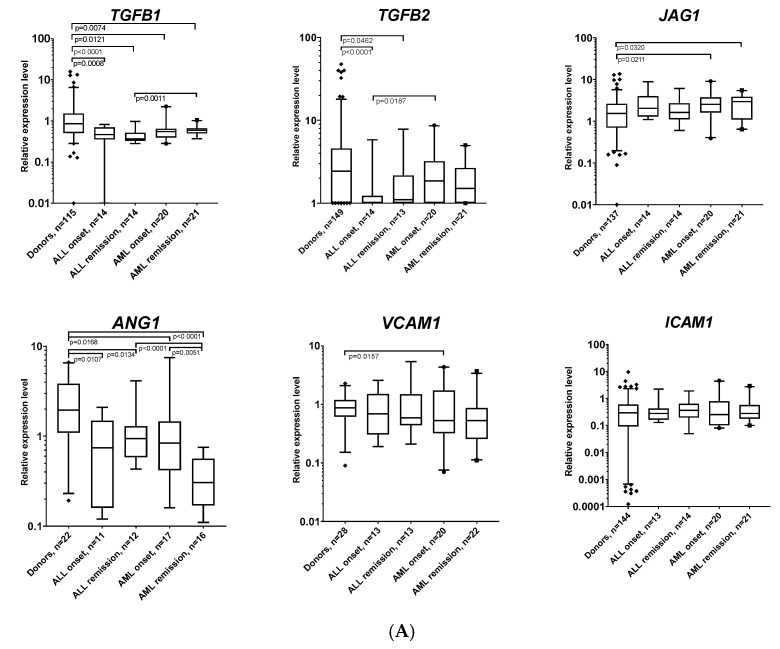

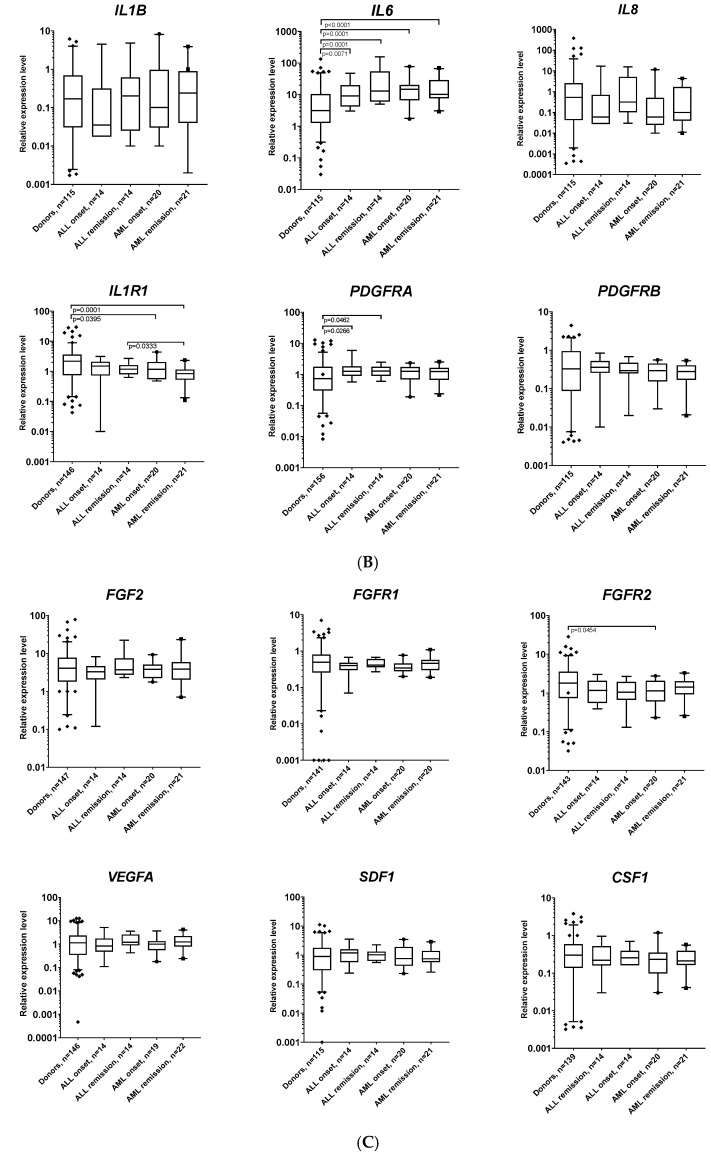

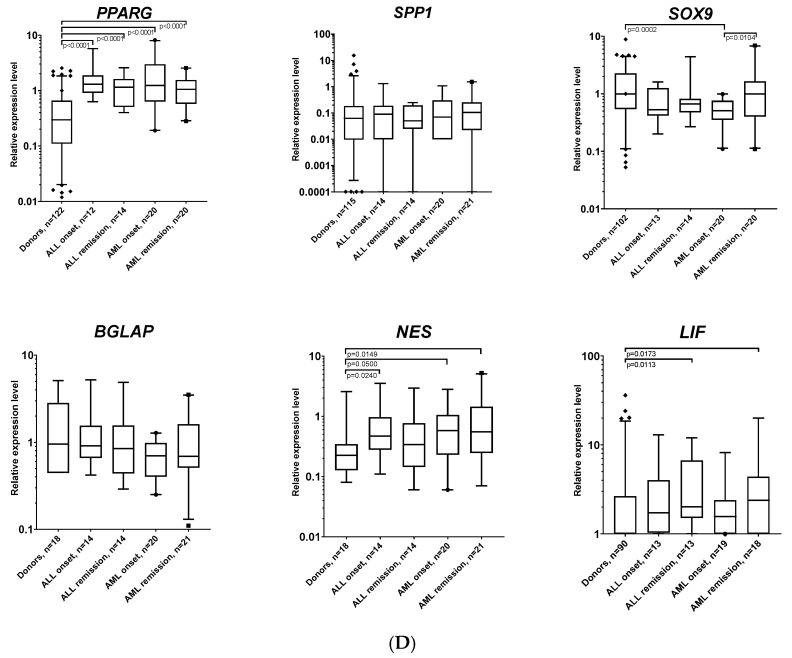
The relative expression levels of genes important for MSC differentiation and function. The box plots’ whiskers cover the 95th percentile. Statistical analysis in all panels was performed using the Mann–Whitney test. The analyzed genes are grouped by the functions of their proteins: (**A**) factors involved in HSC regulation; (**B**) cytokines and cytokine receptors; (**C**) growth factors, chemokines and their receptors; (**D**) factors involved in MSCs’ differentiation.

The relative expression levels of genes encoding factors important for the maintenance of hematopoietic stem cells were analyzed (Figure 2A). *TGFB1* expression was significantly reduced in AL-MSCs at the onset and in remission compared to D-MSCs. *TGFB2* expression was significantly reduced in ALL-MSCs at the onset and in remission compared to D-MSCs and AML-MSCs at the onset of the disease. *JAG1* expression was significantly increased in AML-MSCs at the onset and in remission compared to D-MSCs. *ANG1* expression was significantly reduced in AL-MSCs both at the onset and in remission compared to D-MSCs. In ALL-MSCs in remission, *ANG1* expression increased compared with ALL onset, whereas in AML-MSCs, it decreased in remission compared with AML onset. *VCAM1* expression was significantly reduced only in AML-MSCs at the onset. No changes were detected in the expression of *ICAM1*, *IL1B*, *IL8*, *PDGFRB*, *FGF2*, *FGFR1*, *VEGFA*, *SDF1* and *CSF1* (Figure 2A–C).

*IL6* expression was increased in all AL-MSC groups at the onset and in remission compared to D-MSCs. *IL1R1* expression was decreased in patients with AML at the onset and in remission. In remission, it was significantly lower in patients with AML than in patients with ALL. *PDGFRA* expression was increased in patients with ALL at the onset and in remission compared to donors (Figure 2B). FGFR2 expression was only decreased at the onset of AML (Figure 2C).

Analysis of the expression of genes associated with MSC differentiation revealed an increase in the relative level of *PPARG* expression in all patients with AL at the onset and remission of the disease. No changes in the genes involved in bone differentiation—*SPP1* and *BGLAP*—were detected. A decrease in the expression of the cartilage differentiation marker gene—*SOX9*—was detected in patients with AML at the onset of the disease compared to donors. Its level increases upon achieving remission. The level of *NES* expression is increased in patients with AL at the onset of the disease and remains elevated in patients with AML upon achieving remission. *LIF* expression is significantly increased in patients with AL in remission compared to donors (Figure 2D).

It is known that gene transcription does not always lead to translation and protein formation. MSCs’ secretory function is crucial for hematopoiesis reguation as well as interaction with malignant cells, so the study of MSCs’ secretomes might be important.

### 2.2. Comparison of AL-MSC and Donor-MSC Secretomes

In MSCs’ secretomes, a total of 2832 proteins were identified. The D-MSC secretome lacked 525 proteins identified in AL-MSCs. AL-MSCs in remission lacked 1194 proteins compared to D-MSCs and AL-MSCs at the onset of the disease.

**Figure 3:**
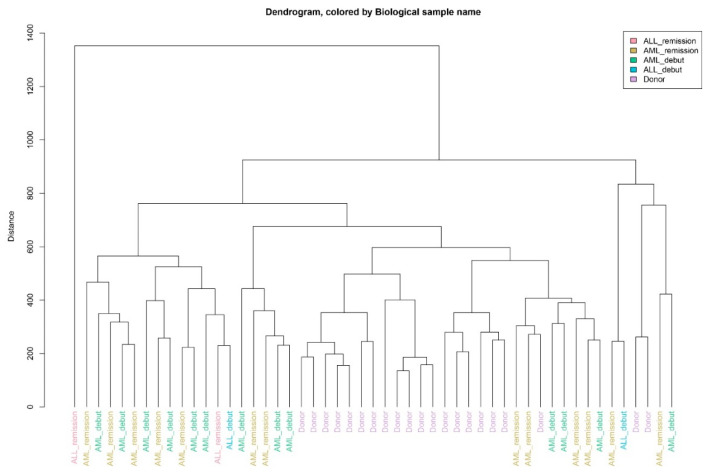
Dendrogram demonstrating the secretome samples clustering.

The secretomes of AL-MSCs were significantly different from the D-MSCs’ ones. The donor samples clustered together, and in many cases onset and remission samples clustered close to each other. This fact suggests that there were no pronounced differences between AL-MSC secretomes at the onset and in remission (Figure 3).

**Figure 4:**
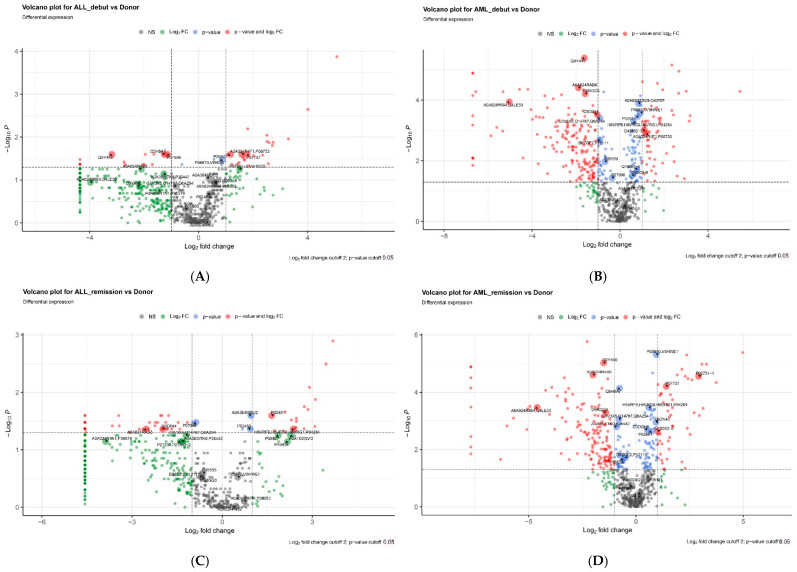
Volcano plots demonstrating the differences between the secretomes. (**A**) ALL onset vs. donors; (**B**) AML onset vs. donors; (**C**) ALL remission vs. donors; (**D**) AML remission vs. donors.

There were fewer significant differences between the secretomes of D-MSCs and ALL-MSCs at the onset and in remission than between the secretomes of D-MSCs and AML-MSCs (Figure 4). Principal component analysis revealed the proteins that were the most different (Figure S1, Table S1, Supplementary Materials).

**Figure 5:**
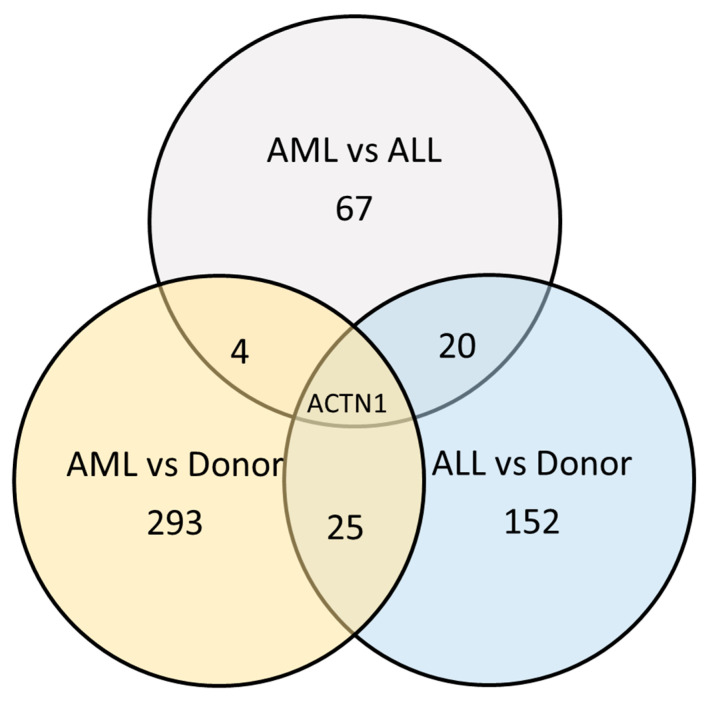
The numbers of proteins that differed between AL-MSCs’ secretome at the onset of leukemia and D-MSCs’ secretome.

AL-MSC secretomes at the onset revealed 116 exclusive secreted proteins. According to the STRING service, those proteins were enriched in proteins related to extracellular space and included CCL5, CXCL3, HLA-A, HLA-C, ACTN1, Serotransferrin (TF), collagens 2 and 4 and keratins. Among those, 98 proteins were exclusive to AML, including metallothionein(MT1E) and NKTR. D-MSCs’ secretome differed from AML-MSCs’ in 293 proteins, and from ALL-MSCs’ in 152 proteins. AML-MSCs’ and ALL-MSCs’ secretomes differed in 67 proteins (Figure 5). The AL-MSCs’ secretomes are more alike than any of them is alike to D-MSCs’.

In AML-MSCs, the secretion of 17 proteins was significantly higher than in D-MSCs, both at the onset and in remission (Table 1). Those 17 proteins are related to cell–cell interaction; among them are insulin-like growth factor binding proteins, proteins linked to phospholipids and cadherin. In AML-MSCs, the secretion of 30 proteins was significantly lower than in D-MSCs, both at the onset and in remission (Table 1). These proteins are involved in inflammation, macrophage differentiation and HSC quiescence maintenance. So, in AML-MSCs, the secretion of proteins involved in normal hematopoiesis regulation was decreased.

D-MSCs and ALL-MSCs differed in 152 proteins. In ALL-MSCs, the secretion of 11 proteins was increased, and the secretion of 6 proteins was decreased (Table 2). Those proteins are related to extracellular matrix binding and exosomes.

Compared to D-MSCs, 719 proteins were absent in AML-MSCs’ secretomes, and 1197 proteins were absent in ALL-MSCs’ secretomes; of those, 696 proteins matched. The most important proteins that were only found in D-MSCs’ secretome included ALPL, BAX, CFHR1, CD276, CD74, CD14, CRP, FGF7, FLT4, GDF15, GDF2, GDF6, CTCSK, HLA-A, HLA-B, HLA-C, IL4I1, KIT, ELN, FABP4, FABP3, FABP1, LYZ, MCAM, MMP2, MMP9, MMP13, MMP19, NCAM, NOTCH2, NOTCH3, NPM3, NQO2, NT5E, OSTF1, PDGFC, PGF, POSTN, S100A7, S100A8, S100A9, S100A12, SERPINA1, SERPINA10, SPP2, TGFB3, VEGFB, PTGES3, SF3A1, SF3A2 and VWA1. Those proteins are involved in VEGF regulation and angiogenesis, as well as in the BMP signaling pathway, thus participating in hematopoiesis, morphogenesis, growth factor response and immune effector processes. In addition, ALL-MSCs’ secretome lacked proteins essential for endothelial proliferation, the regulation of platelet activation, response to vitamin D, the chemotaxis and migration of blood cells, the regulation of growth and other vital functions: CCL5, CTSB, CTSC, CTSF, CXCL1, CXCL8, EGFR, FABP5, ICAM1, FGFR1, FTL, HGF, HLA-DQ, IGF2, IGF2R, MET, SERPINA3, LTBP4, PDGFRB, SF3B1, SPP1, TGFB1, TGFB2 and YAP1.

Our data demonstrate impairment of AL-MSCs’ secretory function. However, it is not the only function of the MSCs. Proteome analysis could provide integrated information to further uncover the bone marrow microenvironment alterations in AL.

### 2.3. Comparison of AL-MSC and Donor-MSC Proteomes

A total of 6715 proteins were identified in the MSCs’ proteome. Of those, 2384 were not found in donor MSCs; 1435 were not found in AML-MSCs at the onset; 1782 were not found in ALL-MSCs at the onset; 991 were not found in AML-MSCs in remission; 1849 were not found in ALL-MSCs in remission.

Of those, 495 proteins were exclusive for AL-MSCs at the onset. Those included proteins belonging to the MHC complex: HLA-A, HLA-B, HLA-C, HLA-DRA, HLA-DPB and HLA-E. JAG-1 and PTGS were among these proteins, too. According to the STRING service, those proteins were enriched in proteins involved in viral infections, such as Epstein–Barr virus (KEGG pathways database, strength 0.49, FDR = 0.0308) and human papillomavirus (KEGG pathways database, strength 0.41, FDR = 0.0276) infections.

**Figure 6:**
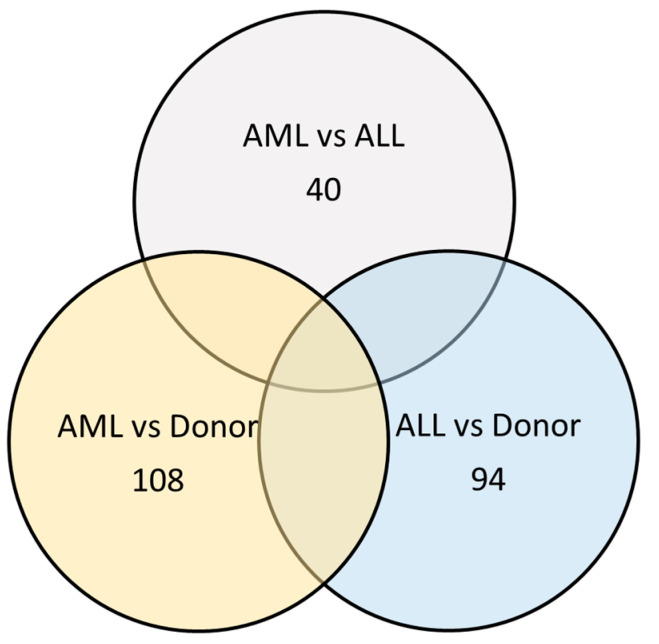
Numbers of proteins that differ in the proteome of AL-MSCs at the onset compared to donors and between AML-MSCs and ALL-MSCs.

AML-MSCs at the onset contained 205 exclusive proteins. The proteome of D-MSCs differed from the proteome of AML-MSCs by 108 proteins and from the proteome of ALL-MSCs by 94 proteins (Figure 6).

**Figure 7:**
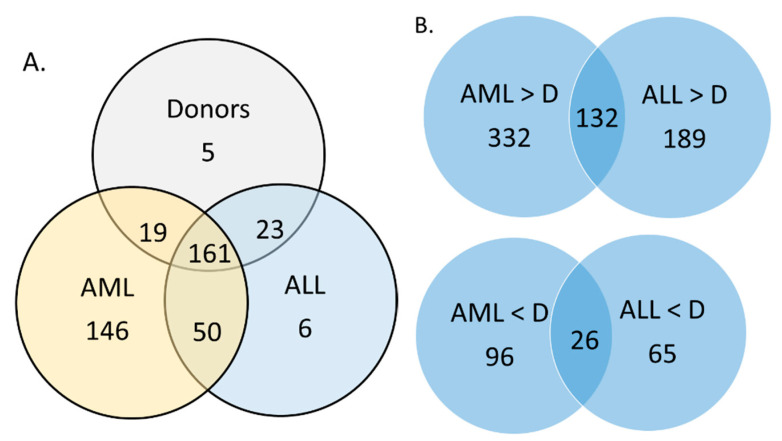
Numbers of proteins that differ in the proteome of AL-MSCs in remission compared to D-MSCs and between AML-MSCs and ALL-MSCs. (**A**) Numbers of proteins that are common or unique for the proteome of the analyzed MSCs groups. (**B**) Numbers of proteins that differ significantly between the groups.

The proteomes of AML-MSCs and ALL-MSCs differ by 40 proteins. The proteomes of AL-MSCs differ less from each other than each of them differs from donors (Figure 7).

AML-MSCs’ proteomes in remission, compared to D-MSCs’, contain 182 extra proteins associated with the processes of translation, transport and metabolism of protein molecules. The majority of these proteins are associated with the process of binding, and they localize in organelles such as ribosomes, mitochondria and the Golgi apparatus, as well as vesicles (Table S2, Supplementary Materials). Of those proteins, 46 were present in AML-MSCs, both at the onset and in remission. These are mainly proteins related to RNA binding and intracellular organization (Table S3, Supplementary Materials).

In contrast to AML, in ALL-MSCs in remission, only 79 proteins differed from the D-MSCs’ proteome, which mainly affect the organization of various tissues (Table S4, Supplementary Materials), and, according to the STRING service analysis, these proteins do not form significantly more connections than a random set, so there likely were not meaningful clusters. ALL-MSCs had 31 proteins in common at the onset and in remission that were not detected in D-MSCs (Table S5, Supplementary Materials).

AML-MSCs in remission expressed higher levels of 464 proteins compared to D-MSCs. Those proteins were associated with both glycolysis and mitochondrial metabolism, the regulation of transcription and translation, osteogenic differentiation and other processes. Among them, for example, are proteasome proteins (thus enhancing the potential of MSCs’ immunological function), CD44 (associated with migration and intercellular interactions, playing an important role in microenvironment organization), POSTN (important for cell adhesion, enhancing the expression of VEGF family growth factors and involved in tumor cell sustenance [8]), differentiation-associated proteins (NES), the annexins ANXA1 and ANXA2, and others (Table S6, Supplementary Materials). AML-MSCs in remission expressed lesser levels of 119 proteins associated with keratins, adhesion and differentiation compared to D-MSCs (Table S7, Supplementary Materials).

ALL-MSCs in remission expressed higher levels of 319 proteins compared to D-MSCs. These proteins are associated with mitochondrial metabolism, intracellular transport and matrix organization (Table S8, Supplementary Materials). ALL-MSCs in remission had lower levels of 86 proteins associated with angiogenesis and endothelial function, adhesion and the organization of the extracellular matrix (Table S9, Supplementary Materials).

The proteome of AL-MSCs in remission contained 132 proteins that were increased compared to D-MSCs both in AML-MSCs and ALL-MSCs. These proteins are associated with cellular metabolism, protein–protein interactions and exosomes (Table S10, Supplementary Materials). At the same time, 23 proteins were decreased compared to D-MSCs both in AML-MSCs and ALL-MSCs. These proteins are associated with hematopoiesis regulation via exosomes and the extracellular space, as well as MHC class 1 (Table S11, Supplementary Materials). In the proteomes of AL-MSCs in remission, five proteins were not detected: ALDH3B1, CFAP46, HLA-A, PADI2 and STBD1. In contrast, 51 proteins were not detected in D-MSCs’ proteomes but found in both AML-MSCs and ALL-MSCs. According to STRING analysis, this set of ptoteins does not contain significantly more interaction than a random selection (Table S12, Supplementary Materials).

When comparing the proteomes of AML-MSCs at the onset and in remission, the expression of 269 proteins increases in remission, while that of 141 proteins decreases (Tables S13 and S14, Supplementary Materials). Proteins associated with the organization of the nucleus and vesicles and with transport increased in remission. Proteins associated with mitochondria, exosomes and the organization of the extracellular space decreased in remission.

When comparing the proteomes of ALL-MSCs at the onset and in remission, the expression of 49 proteins increased in remission, while that of 75 proteins decreased (Tables S15 and S16, Supplementary Materials). Proteins associated with gene expression and metabolic processes were increased in remission. Proteins associated with metabolic processes (glycolysis in particular) and transport were decreased in remission. The ALL-MSCs demonstrated multidirectional changes in metabolic processes, indicating dysregulation of the MSCs.

### 2.4. Comparison of Secretomes and Proteomes

**Figure 8:**
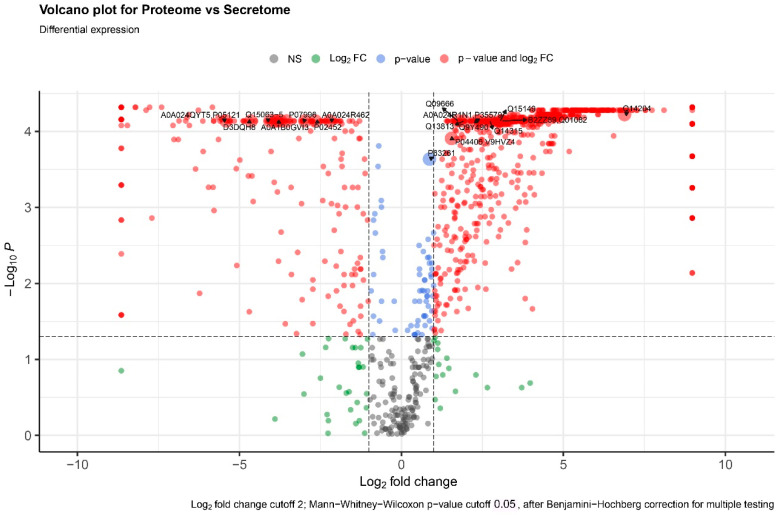
Volcano plot of proteins that differ between proteomes and secretomes.

The proteome and secretome of MSCs differ from each other. Not all proteins detected in the proteome are secreted by the cells. Moreover, not all secreted proteins were detected in the proteome (Figure 8).

In order to compare secretomes with proteomes, for each protein found, in both the secretome and proteome, a ratio of the average protein abundance in the secretome to the average protein abundance in the proteome was determined. These ratios were then compared between MSC groups.

This ratio was higher in AML-MSCs at the onset than in D-MSCs for 21 proteins (Table 3). These proteins are related to the extracellular matrix, the regulation of apoptosis and the regulation of innate immunity. This ratio was lower in AML- MSCs than in D-MSCs for another 21 proteins. These proteins are related to extracellular vesicles and exosomes, as well as intercellular contacts.

The ratio also differed between D-MSCs and AML-MSCs in remission: for 21 proteins, it was higher in AML-MSCs, and for 29, it was higher in D-MSCs. In AML-MSCs, the ratio was higher for proteins associated with the response to stress and various other stimuli, the inhibition of various biological processes and the activation and aggregation of platelets, as well as the extracellular matrix and extracellular vesicles. In D-MSCs, the ratio was higher for proteins associated with the cytoskeleton, cell adhesion, secreted granules and vesicles.

For 9 proteins, the ratio decreased in remission compared to onset, and for 15 proteins, it increased. The ratio decreased for proteins involved in the construction of the extracellular matrix, cell adhesion and the formation of extracellular exosomes. The ratio increased for proteins associated with extracellular vesicles, exosomes and the matrix. The same processes were disturbed in the secretome as in the proteome.

The obtained results indicate dramatic differences in the transcriptome, proteome and secretome of MSCs from the bone marrow of patients with AL at the onset of and in remission from the disease and those of MSCs from the bone marrow of healthy donors.

## 3. Discussion

Nowadays, the tumor microenvironment is considered one of the main players in cancer development and progression. The leukemic microenvironment is highly complex and includes MSCs, their progeny and a large list of extracellular matrix proteins and soluble factors secreted by AL-MSCs. We have studied one of the microenvironment elements—MSCs—in vitro. It is important to note that MSC characteristics vary widely depending on age, sex and somatic status, so each patient group required its own matched donor group.

During AL development, stromal progenitor cells change, and these changes affect proliferation, differentiation, regulatory processes and secretory activity. A decrease in the proliferative potential of MSCs and their ability to grow in culture in AL was observed by us and other researchers [9,10,11].

The proliferation and survival of MSCs depend on a large number of growth factors secreted by the MSCs themselves and those coming from outside. The most pronounced decrease in MSCs’ cumulative production was seen at the onset of AL. It coincided with a significant decrease in the secretion of FGFR1 by AML-MSCs and its absence in ALL-MSCs’ secretomes. FGFR1 is a growth factor receptor that may play a role in MSC proliferation. In the secretome of AL-MSCs, unlike D-MSCs’, TGFB3, FGF7, KIT, PDGFC, PGF and VEGFB were not detected. In ALL-MSCs’ secretomes, in addition, HGF and IGF2 were not found. AML-MSCs secreted significantly less PDGFA than D-MSCs (Log2FC = −3.61, *p* = 0.017).

Growth factors affect MSCs through the following receptors: PDGFR, EGFR, FGFR, c-Met and LRP5 [12]. In AL-MSCs’ proteomes, there was a tendency for the content of PDGFRA to decrease, and PDGFRB was significantly reduced in the secretome of AML-MSCs at the onset (Log2FC = −4.12, *p* = 0.0009). EGFR was absent in the secretome of ALL-MSCs. EGFR was detected in the secretomes of AML-MSCs and D-MSCs, but no significant differences were found between these groups. FGFR1, as mentioned above, was significantly reduced in AML-MSCs’ secretomes and was not found in ALL-MSCs’. C-Met (MET) was absent in AL-MSCs’ secretomes but was detected in D-MSCs’. According to GO Process analysis, Wnt pathway signaling was reduced in the secretomes and proteomes of AL-MSCs compared to those of D-MSCs. Our data correspond with other studies [13,14]. It was identified that leukemic cells prime bone marrow stromal cells towards osteoblast lineage and promote drug resistance. This biased differentiation of stroma is accompanied by the dysregulation of the canonical Wnt signaling pathway. The inhibition of Wnt signaling in stroma reversed drug resistance in leukemic cells [15]. At AL’s onset, we observed a decrease in the production of growth factors and their receptors, which are important both for the proliferation of MSCs and for maintaining hematopoiesis.

Upon achieving remission, the growth parameters of AL-MSCs improved but never reached D-MSCs’ level. This may be due to the fact that the secretion of most growth factors and their receptors remained reduced. For example, in AML-MSCs’ secretomes, PDGFA (Log2FC = −3.1, *p* = 0.02) and PDGFRB (Log2FC = −3.55, *p* = 0.002) were still significantly reduced compared to D-MSCs.

Many studies indicate that the differentiation potential of MSCs changes during the development of leukemia. However, the direction of reported differentiation disturbance is controversial. Some studies show that AL-MSCs’ differentiation potential is skewed towards osteogenesis, leading to bone marrow adipocyte deficiency [11,16]; others suggest enhanced adipogenesis [17,18].

The mRNA expression analysis conducted in this work implies that AL-MSCs were more predisposed to adipogenic differentiation, as we noted a dramatic increase in *PPARG* expression in all AL-MSC groups, as well as a decrease in *SOX9* expression in AML-MSCs at the onset. Transcription analysis did not reveal any alterations in the studied bone differentiation markers: *SPP1* and *BGLAP*. However, these changes did not translate to proteome nor secretome levels. Greenbaun and colleagues attempted to address such a situation and suggested the term “translatome” to describe interrelated mRNA transcripts and protein populations [19]. This discrepancy between transcriptomics and proteomics can be ascribed to several reasons. The translation level does not directly correlate with the transcription level due to post-transcriptional regulation mechanisms. In addition, some of the proteins might be small enough in size or quantity to be lost in the process of mass spectrometry sample preparation.

Of the differentiation markers studied at the mRNA level, only SPP1 protein presence was found in the secretomes of D-MSCs and AML-MSCs. AML-MSCs in remission secreted significantly more SPP1 than D-MSCs (Log2FC = 2.94, *p* = 0.031).

Alkaline phosphatase (ALPL) was significantly increased in the proteomes of ALL-MSCs at the onset compared to both D-MSCs (Log2FC = 3.57, *p* = 0.004) and ALL remission (Log2FC = 1.4, *p* = 0.02). AML-MSCs in remission secreted more ALPL compared to D-MSCs (Log2FC = 5.06, *p* = 0.004) and AML-MSCs at the onset (Log2FC = −1.6, *p* = 0.02).

Periostin (POSTN) is a multifunctional extracellular component that takes part in regulating osteogenesis, cell–matrix interaction and cell–cell crosstalk. Moreover, POSTN overexpression promoted B-ALL progression [20]. POSTN was detected in both the proteomes and secretomes of all studied MSC groups. It was increased in the proteome of ALL-MSCs at the onset compared to D-MSCs (Log2FC = 0.38, *p* = 0.046), but it was dramatically reduced in secretome (Log2FC = −2.74, *p* = 0.04). In remission, POSTN was increased in the proteome of ALL-MSCs compared to D-MSCs (Log2FC = 0.82, *p* = 0.05), and the decrease in secretion was not statistically significant. In AML-MSCs at the onset, the secretion of POSTN was slightly elevated (Log2FC = 0.13, *p* = 0.01) compared to D-MSCs. In AML remission, POSTN was increased compared to D-MSCs in both the proteome (Log2FC = 1.81, *p* = 0.001) and secretome (Log2FC = 0.38, *p* = 0.001).

Our proteomics data suggest a predisposition to the osteogenic potential of AL-MSCs.

A positive correlation of POSTN and CCL2 expression was noted in Ma’s work [20]. The CCL2 chemokine is a macrophage regulator that can also activate tumor cell growth and proliferation [21]. In our study, CCL2 secretion was significantly increased in AML-MSCs compared to D-MSCs both at the onset (Log2FC = 2.4, *p* = 0.006) and in remission (Log2FC = 2.96, *p* = 0.0001), but there were no differences in ALL-MSCs. In AL, the interaction of POSTN and CCL2 is disturbed.

Aggrecan (ACAN) is a main component of cartilage extracellular matrix, and it is required for chondrocytic differentiation [22]. Aggrecan secretion was significantly lower in AML-MSCs compared to donors both at the onset (Log2FC = −2.8, *p* = 0.003) and in remission (Log2FC = −3.87, *p* = 0.005). Chondroitin sulfate proteoglycan 4 (CSPG4) was also lower in the secretomes of AML-MSCs compared to D-MSCs both at the onset (Log2FC = −3.2, *p* = 0.001) and in remission (Log2FC = −2.85, *p* = 0.006). Another chondrocytic marker, SOX9, wan not detected by mass spectrometry, but *SOX9* gene expression was decreased in AML-MSCs at the onset compared to D-MSCs. Alshenibr et al. [23] and Tashkandi et al. [24] works had shown that LOXL2 overexpression led to increase in expression of ACAN, CSPG4, SOX9 and COL2A1, and inhibited chondrocyte apoptosis. TGFB1 stimulation, in turn, upregulated LOXL2. Decrease in the expression of all those genes and proteins suggests dramatic alterations in chondrogenic potential of AML-MSCs.

AML-MSCs’ proteome and secretome contained increased abundance of galectin-3 (LGALS3), which can support tumor cell survival and may be involved in AL relapse, and also regulates osteogenic and adipogenic differentiation [25]. Our data support the upregulation of both adipogenic and osteogenic AL-MSCs’ potential compared to D-MSCs and suggest disturbed differentiation of these cells.

Nestin (NES) is a marker of multipotent cells [26]. Additionally, Nes(+) cells were shown to be vasculature-associated early cells in the osteoblast and endothelial lineage [27]. Nestin production was dramatically changed in all AL-MSCs. *NES* gene expression was significantly higher in AL-MSCs at all studied time points than in D-MSCs. The NES amount in the proteome was significantly higher, too, in AL-MSCs.

These changes may be important for the regulation of hematopoiesis. It was shown that Nes(+) MSCs secreted lower levels of HSC-supporting factors, including SCF, CXCL12 and ANG1 [28,29]. This is backed by AL-MSC secretome and proteome analyses. COL1A1 and COL1A2 are involved in the stromal regulation of hematopoiesis. Col1a1 regulates Ang-1/Tie2 interactions in MSCs and HSCs [30]. COL1A1 secretion was decreased in AML-MSCs at the onset (Log2FC = −0.1, *p* = 0.047) and in ALL-MSCs in remission (Log2FC = −2.39, *p* = 0.031) relative to D-MSCs. COL1A1 was decreased in ALL-MSCs’ proteome compared with AML-MSCs’ (Log2FC = −0.42, *p* = 0.04). COL1A2 was involved in HSC proliferation and differentiation [31]. The level of COL1A2 was reduced in the proteome of AML-MSCs at the onset (Log2FC = 0.26, *p* = 0.036) and remission (Log2FC = 0.48, *p* = 0.006) compared to D-MSCs. COL1A2 was also lower in the proteome of ALL-MSCs in remission than in D-MSCs’ (Log2FC = −0.26, *p* = 0.039). Moreover, in the secretome of ALL-MSCs in remission, COL1A2 is reduced relative to D-MSCs (Log2FC = −2.37, *p* = 0.007) and AML-MSCs in remission (Log2FC = −1.77, *p* = 0.038). Changes in the composition of the extracellular matrix may disturb HSC regulation in AL patients.

*ANG1* expression levels varied between the studied groups, but ANG was not detected by mass spectrometry in neither proteome nor secretome samples. *ANG1* expression was lower in all studied AL-MSCs groups than in D-MSCs. ANG1 decrease may affect MSCs’ interactions with HSCs and alter MSC differentiation potential, skewing it towards adipogenesis [32,33].

TGFB1 is one of the most important proteins that maintain HSCs in quiescent state [34,35]. *TGFB1* gene expression was decreased in all AL-MSCs compared to D-MSCs. TGFB1 was decreased in the secretomes of AML-MSCs compared to D-MSCs, both at the onset (Log2FC = −4.6, *p* = 0.0004) and in remission (Log2FC = −4.08, *p* = 0.0007), and it was not detected in ALL-MSCs. Additionally, in AML-MSCs, there was a decrease in the secretion of TGFB large latent complex proteins—LTBP1 and LTBP2 (Table 1)—that keep TGFB inactive [36]. Contrary to that, the TGFB1 protein was not found in D-MSCs’ proteomes and was exclusively present in the proteomes of patients at the onset of AML. It is possible that while AML-MSCs produced TGFB1, its secretion was inhibited. TGFB2 and TGFB3 modulate growth factors involved in myelopoiesis [37,38]. TGFB2 was not detected in ALL-MSC secretomes both at the onset and in remission, and TGFB3 secretion was only detected in D-MSCs. In the proteome, TGFB2 was only found in a single donor’s sample. Put together, these data suggest that the HSCs of AL patients could be activated, which leads to their exhaustion and may negatively influence hematopoiesis restoration in long-term remission.

VCAM1 was decreased both in terms of gene expression and secretome levels in AML-MSCs at the onset and in remission, as well as in ALL-MSCs’ proteome in remission. In ALL-MSCs at the onset and in remission, VCAM1 secretion was the only one to decrease compared to D-MSCs. This corresponds with other authors’ findings [10,39]. VCAM-1 is an adhesion molecule; the decrease in its production disrupts intercellular interaction and, consecutively, the functionality of AL-MSCs.

Jagged-1 (JAG-1) is a ligand of Notch family receptors, activating downstream gene expression [40]. *JAG1* expression was higher in AML-MSCs than in D-MSCs; the JAG-1 protein was exclusively found in the proteomes of AML-MSCs at the onset. However, JAG-1 secretion was only detected in D-MSCs. A multitude of studies links JAG-1 overexpression to malignancy and other pathological processes [41]. An increase in JAG1 expression and an increase in its presence in AML-MSCs’ proteome coupled with decreased secretion implies disrupted processing of this protein. Jagged-1 was most likely secreted as a part of exosomes, whose secretion was altered in AML-MSCs. It is possible that JAG-1 surface expression causes abnormally high Notch signaling in neighboring cells and leads to an abnormal HSC phenotype. It was recently shown that galectin-3 (LGALS3) is implicated in regulating Jag1–Notch interaction and function, since it is able to competitively bind to Jag-1 [42]. According to our data, Galectin-3 was increased in both the secretomes and proteomes of AML-MSCs at the onset and in remission compared to D-MSCs. We may suppose that a Jag-1 increase leads to a Galectin-3 increase as a negative feedback loop.

LIF is a pleiotropic growth factor [43]. *LIF* gene expression increased in AL-MSCs in remission compared to D-MSCs. No significant differences were found in LIF secretion, and it was not detected in the proteome. Leukemia and chemotherapy disrupt bone marrow niches [44]. Blood vessels and endothelium-derived stromal cells are crucial niche components [45]. *LIF* upregulation in remission might happen due to its ability to induce MSCs’ angiogenic potential [46]. On the other hand, LIF overexpression is frequently noted in various malignancies. It is possible that LIF plays a role in oncogenesis and relapse [47]. However, an increase in LIF protein level was not detected, so perhaps there was no restoration of MSCs’ angiogenic function in remission.

*IL6* gene expression was higher in AL-MSCs at the onset and in remission than in D-MSCs. The IL6 protein was detected in the secretomes of all the MSC groups at a similar level. IL6 was not detected in any of the proteome samples. IL6 suppresses the differentiation of normal HSCs, leading to cytopenia and anemia [48]. An increase in mRNA expression should not impact MSC functionality, considering the lack of alterations at the proteome and secretome levels.

The CXCL12 cytokine is of great importance for the regulation of HSCs and their progeny [36,49]. Moreover, the interaction of CXCL12 with the CXCR4 receptor plays an important role in the pathogenesis of hematological tumors [50]. Its secretion was significantly increased in ALL-MSCs at the onset (Log2FC = 1.77, *p* = 0.0001) and AML-MSCs in remission (Log2FC = 1.53, *p* = 0.044) compared to D-MSCs. In ALL remission, the differences in CXCL12 secretion between ALL-MSCs and D-MSCs disappeared. It was shown that the plasma level of CXCL12 is strongly elevated at ALL’s onset [51], which is consistent with our results. However, other studies indicate a drop in its concentration in the bone marrow in ALL [52,53]. Considering the absence of differences in the levels of gene expression and protein production, changes occur in the regulation of protein secretion.

Macrophage colony-stimulating factor (M-CSF, CSF1) is a locally secreted growth factor that stimulates the proliferation and differentiation of macrophages and osteoclasts [54]. AML-MSCs at the onset secreted less CSF1 than D-MSCs (Log2FC = −2.79, *p* = 0.0007), and its secretion remained reduced in remission (Log2FC = −2.71, *p* = 0.0004). This protein was not detected in the proteome. CSF1 secretion is associated with the maintenance of normal hematopoiesis and bone remodeling [55]. A CSF1 deficiency found in the AML-MSCs secretome could negatively affect osteogenesis and hematopoiesis. The CSF1 receptor, CSF1R, is considered a potential target for precision therapy in the treatment of various malignancies, including AML. In this case, the aim is to block the paracrine signals from the tumor microenvironment [56].

Macrophage migration inhibitory factor (MIF) is constitutively present in several cell and tissue types and is secreted in response to acute inflammation [57]. On the other hand, MIF enhances MSCs’ immunosuppressive properties in various models, such as myocardial repair [58] and allergic asthma [59]. MIF was detected in the proteome of MSCs of all studied groups. Its content was reduced in ALL-MSCs compared to D- MSCs (Log2FC = −0.95, *p* = 0.05). In the AML-MSCs proteome, an insignificant decrease was observed at the onset. MIF was not detected in the secretome of D-MSCs and ALL-MSCs in remission. Thus, healthy MSCs do not secrete MIF, while the MSCs of patients with both AML and ALL at the onset do secrete it. At the same time, in ALL-MSCs in remission, MIF secretion disappeared, while in AML-MSCs in remission, it decreased.

In the proteome and secretome of AL-MSCs, a change in proteins associated with leukemia was observed. Among these proteins was FLOT1 (Flotillin-1), a participant and marker of lipid raft formation in the plasma membrane. FLOT1 is involved in many cellular processes, including adhesion, actin cytoskeleton reorganization, endocytosis, phagocytosis and signaling, and its overexpression has been described in many malignancies [60]. An increase in FLOT1 expression in AML blasts compared to normal hematopoietic cells was described, and such an increase correlated with unfavorable prognosis [61]. According to our data, the level of FLOT1 was elevated compared to D-MSCs in AML-MSCs’ proteome at the onset (Log2FC = 1.38, *p* = 0.004) and in remission (Log2FC = 1.59, *p* = 0.027). In ALL-MSCs at the onset, the FLOT1 level was significantly higher than in remission (Log2FC = 0.42, *p* = 0.02). Its presence in the secretome was not detected, despite the fact that FLOT1 is described as a marker of cellular exosomes [62]. The content of nuclear proteins and transcription factors associated with AL was multidirectionally changed in AL-MSCs’ proteome. These proteins include CCNH, PRKDC, PML, EML4, LMNB1, SIN3A and CCAR1. The following splicing proteins were also altered: SNRPB2, SNRNP70, RBM10, LSM4 and SRSF2.

The proteome and secretome of AL patients’ MSCs differed from those of healthy donors. For example, the secretome of D-MSCs contained growth factor FGF7, which is involved in osteogenesis and induces keratin synthesis [63]. FGF7 was absent in the MSC secretomes of AL patients, both at the onset and in remission. The proteomes and secretomes of ALL-MSCs were significantly different from those of AML-MSCs. Changes in MSC proteomes and secretomes did not always coincide. The combined analysis of proteomes and secretomes sometimes revealed specific differences, such as in the study of Toll-like receptors in macrophages in vitro [64] or the characterization of insulin-producing cells [65]. Not all secreted proteins are detected in the cell proteome. Out of 12,917 intracellular proteins and 3123 membrane-associated proteins, only 664 coincide with known secreted proteins; the rest are not detected in secretomes (Human Protein Atlas, ver. 23.0). The ratio between proteome and secretome proteins varies greatly in donor and patient MSCs and may have physiological significance. Proteins detected in the proteome and secretome of patients’ MSCs at the onset of the disease differ from those detected in remission.

In AL, changes occur not only at the level of hematopoietic cells but also at the global organismal level. In this study, both changes common to AML and ALL and changes dependent on nosology were detected. Differences between ALL and AML may be associated with damage to MSCs caused by blast cells and differences in the received treatment. The functional changes in the stromal microenvironment studied in this work can be important for understanding the pathogenesis and progression of AL and for discovering new therapeutic targets.

## 4. Materials and Methods

### 4.1. Patients

This study included MSCs obtained from the bone marrow of AML and ALL patients at the onset and from the same patients in remission from the disease in the period from the beginning of the year 2021 to the end of the year 2023. MSCs from the bone marrow of healthy donors were used as controls (Table 4). Healthy donors’ bone marrow samples were obtained at the time of exfusion, and AL patients’ bone marrow samples were obtained at the time of the diagnostic punctions. MSCs were isolated immediately after obtaining a bone marrow sample (no more than 2 h after sample collection). All work was conducted in accordance with the Declaration of Helsinki (1964). This study was approved by the local ethics committee, and the donors and patients provided written informed consent.

### 4.2. MSCs

The MSCs were expanded as described [66].

The MSCs were derived from 2–8 mL of bone marrow. To separate the mononuclear cells, the bone marrow was mixed with an equal volume of alpha-MEM (ICN, Laval, QC, Canada) containing 0.2% methylcellulose (1500 cP, Sigma-Aldrich, St. Louis, MO, USA). After 40 min, most of the erythrocytes and granulocytes had sedimented, while the mononuclear cells remained in the supernatant. The supernatant was aspirated and centrifuged for 10 min at 450× *g*. The cells from the pellet were resuspended in a standard culture medium composed of alpha-MEM supplemented with 10% fetal bovine serum (HyClone, Logan, UT, USA), 2 mM L-glutamine (ICN, USA), 100 U/mL penicillin (Synthesis, Moscow, Russia) and 50 mkg/mL streptomycin (BioPharmGarant, Vladimir, Russia). The cells (3 × 10^6^) were cultured in T25 culture flasks (Corning-Costar, New York, NY, USA). After reaching confluency, the cells were washed with 0.02% EDTA (ICN, USA) in a physiological solution (Sigma-Aldrich, USA) and then detached by 0.25% trypsin (ICN, USA) treatment (Passage 0). For expansion, the cells were seeded at 4 × 10^3^ cells per cm^2^ of flask growth area. The cultures were maintained at 37 °C and 5% CO_2_. The number of harvested cells was counted directly; cell viability was determined by trypan blue dye exclusion staining.

Upon reaching confluency, the culture may retain some macrophages and other bone marrow cells. After passaging the culture, non-MSCs are eliminated. At passages 0, 1 and 2, immunofluorescence analysis of the purity of the MSC population was performed as described in previous studies [67]. For expression analysis, MSCs after the first passage were used, and the secretome and proteome were analyzed at the third passage.

### 4.3. Relative Level of Gene Expression Analysis

#### 4.3.1. RNA Isolation

To isolate RNA, the cells of the first passage (10^5^–4.5 × 10^5^ cells) were centrifuged at 300× *g*. The pellet was washed with 1 mL of phosphate buffer and centrifuged at 300× *g*. Next, 400 µL of TriZol (Ambion by Life Technologies, Waltham, Massachusetts, USA) was added to the pellet. Samples with TriZol were stored at −70 °C. After thawing, 120 µL of chloroform was added to the samples, after which they were shaken, incubated for 2 min at room temperature and centrifuged for 15 min at 13,500× *g* and 4 °C in a Centrifuge 5424 R (Eppendorf, Hamburg, Germany). The resulting upper phase was transferred into new tubes. Then, 400 µL of isopropanol was added, and the samples were incubated for 10 min at room temperature and centrifuged for 10 min at 13,500× *g* and 4 °C. The pellet was washed with 1 mL of 75% ethanol, vortexed and centrifuged for 5 min at 13,500× *g* at 4 °C. The pellet was left to dry for 5 min at room temperature. Next, 100 µL of DEPC-treated water was added to the pellet and left for 30 min on ice for it to dissolve. After vortexing, 1 µL was taken to measure the amount of extracted RNA. The measurement was carried out on a NanoDrop One device (Thermo Fisher Scientific, Waltham, MA, USA) at a wavelength of 260 nm, and RNA purity was determined by the ratio of 260/280 nm. To the remaining 99 μL of the RNA solution, 10 μL of 3M sodium acetate and 250 μL of 96% ethanol were added. Samples were stored at −20 °C.

#### 4.3.2. cDNA Acquisition

RNA in a mixture of ethanol and sodium acetate was centrifuged for 10 min at 13,500× *g* and 4 °C. After that, the pellet was washed with 1 mL of 75% ethanol, shaken in a vortex and centrifuged for 5 min at 13,500× *g* and 4 °C. The pellet was left to dry for 5 min at room temperature. Next, 1 µL of DEPC-treated water was added per 1 µg of RNA, and the samples were left on ice for 30 min for dissolution. Primers for reverse transcription (T13 primers and random hexamers) were annealed as follows: 2 µL of RNA solution, 1.25 µL of each primer (40 pmol/µL) and 5.5 µL of DEPC-treated water were mixed and incubated in a Tertsik amplifier (DNA-Technology, Moscow, Russia) for 10 min at 70 °C and 10 min at 4 °C. After that, 15 μL of the reverse transcription mix (5.5 µL milliQ water, 5 µL 5× M-MLV reversease buffer (Promega, Madison, WI, USA), 2.5 µL dNTPs mix, 1 µL each RNAsin (Promega) and M-MLV reversease (Promega)) was added, and the samples were incubated in a Tertsik amplifier at 42 °C for 1 h. Finally, 75 μL of milliQ water was added. The samples were stored at −20 °C.

#### 4.3.3. Real-Time PCR

Real-time PCR with Taq-man modification was performed on an AbiPrism Real Time PCR System 7500 device (Thermo Fisher Scientific, USA) in a 96-well plate, and the reaction volume was 25 µL. Each sample was analyzed in triplicate, a positive control (a reference mixture of cDNA) was used to assess the quality of the reaction and correlate the results of different PCRs, and a no template control was included in each plate. Table S17, in the Supplementary Materials, presents primers’ and probes’ sequences. PCR reagents were mixed into a master mix (12.8 µL milliQ water, 3.5 µL 25 mM MgCl_2_ (Thermo Fisher Scientific), 2.5 µL 2.5 mM dNTPs mix, 2.5 µL 10× SmarTaq buffer (Dialat, Moscow, Russia), 1 µL each forward and reverse primers (10 pmol/µL), 0.5 µL fluorescent probe (10 pmol/µL) and 0.2 µL SmarTaq polymerase (Dialat) per reaction). Then, 72 µL of the master mix was placed into the wells of a 96-well PCR plate, and 3 µL of the cDNA solution was added. The samples were mixed and divided into 3 wells of 25 µL to obtain triplicates. PCR was started with 10 min at 95 °C to activate the polymerase; 40 cycles of PCR were performed for the BACT and GAPDH genes, and 45 cycles were performed for the other genes. The cycle parameters were as follows: 15 s at 95 °C + 40 s at 60 °C.

### 4.4. Preparation of MSC-Conditioned Medium

MSCs at passages 2–3 were seeded at 4 × 10^3^ cells per cm^2^ into T175 flasks (Costar, New York, NY, USA). After attaining confluence (3–4 days), the flasks were washed 5 times with phosphate buffer without Ca^2+^/Mg^2+^ (Invitrogen, Waltham, MA, USA) and then cultured for 24 h in RPMI 1640 medium (HyClone, Logan, UT, USA) without serum and phenol red. The conditioned medium was centrifuged at 400× *g* and stored at −70 °C.

### 4.5. Preparation of MSC Samples for Proteome Analysis

After collecting the conditioned medium (see above), the cells were detached from the flask bottom with a cell scrapper and centrifuged for 10 min at 450× *g*, and the resulting dry pellet was stored at −70 °C.

### 4.6. Mass Spectrometry Sample Preparation

A protease inhibitor cocktail (Halt Protease Inhibitor Cocktail, Thermo Fisher Scientific, USA) was added to each sample, which was then centrifuged at 1500× *g* for 10 min to remove debris. Supernatants were immediately frozen and lyophilized to reduce volume. The lyophilizates were resuspended for 30 min in a buffer containing 6 M Gd-HCl, 10 mM TRIS-HCl (pH 8) and 2 mM DTT. To precipitate the insoluble fraction, the solutions were centrifuged at 16,000× *g* for 10 min at 4 °C. Samples were concentrated using a centrifuge filter (Corning Spin-X UF6, Sigma-Aldrich, USA) to replace the buffer. Buffer (8 M urea, 2 M thiourea, 10 mM TRIS-HCl (pH = 8)) was added to the concentrated samples at a ratio of 1:3 and incubated at room temperature for 30 min. Disulfide bonds were reduced with 5 mM DTT at room temperature for 40 min and then alkylated with 10 mM iodoacetamide in the dark at room temperature for 20 min. Alkylated samples were diluted by adding 50 mM NH_4_HCO_3_ solution in a ratio of 1:4. Then, trypsin was added (0.01 μg per 1 μg of protein), and the samples were incubated at 37 °C for 14 h. The reaction was stopped by adding formic acid to a final concentration of 5%. The peptides were desalted using a Discovery DSC-18 (tubes 1 mL, 50 mg) (Sigma-Aldrich, USA), dried in vacuum and stored at −80 °C before analysis. Prior to LC-MS/MS, samples were redissolved in 5% acetonitrile with 0.1% trifluoroacetic acid and sonicated.

### 4.7. LC-MS/MS Analysis

Proteomic analysis was performed on an Orbitrap Q Exactive HF-X (Thermo Fisher Scientific) mass spectrometer equipped with a nano-electrospray (nano-ESI) source and a high-pressure nanoflow chromatograph UPLC Ultimate 3000 (Thermo Fisher Scientific) equipped with a lab-packed reverse-phase (Kinetex C18, 2.4 μm) column (100 mm × 500 mm). The temperature of the column was thermostatically controlled at 60 °C. Samples were loaded in buffer A (0.1% Formic acid) and eluted with a linear (180 min) gradient of 3 to 55% buffer B (0.1% Formic acid, 80% Acetonitrile) at a flow rate of 220 nL/min. Mass spectrometric data were stored during automatic switching between MS1 scans and up to 16 MS/MS scans (topN method). The target value for MS1 scanning was set to 3 × 10^6^ in the range of 390–1400 *m*/*z* with a maximum ion injection time of 45 ms and a resolution of 60,000. The precursor ions were isolated at a window width of 1.4 *m*/*z*. Precursor ions were fragmented by high-energy dissociation in a C-trap with a normalized collision energy of 30 eV. MS/MS scans were saved with a resolution of 15,000 at 400 *m*/*z* and at a value of 2 × 10^5^ for target ions with a maximum ion injection time of 50 ms.

### 4.8. Protein Identification and Bioinformatics Analysis

Raw LC-MS/MS data from the Q Exactive HF mass spectrometer were converted to *.mgf peaklists with MSConvert (version 3, ProteoWizard Software Foundation). For this procedure, we used the following parameters: “–mgf –filter peakPicking true [1,2]”. For thorough protein identification, the generated peak lists were searched with the MASCOT (version 2.5.1, Matrix Science Ltd., London, UK) and X! Tandem (ALANINE(2017.02.01), The Global Proteome Machine Organization) search engines against the UniProt human protein knowledge base with the concatenated reverse decoy dataset. The precursor and fragment mass tolerance were set at 20 ppm and 0.04 Da, respectively. Database-searching parameters included the following: tryptic digestion with one possible missed cleavage, static modification for carbamidomethyl (C) and dynamic/flexible modifications for oxidation (M). For X!Tandem, parameters were chosen that allowed us to quickly check for the acetylation of the N-terminal residue of the protein, the loss of ammonia from the N-terminal glutamine and water from the N-terminal glutamic acid. The resulting files were processed in Scaffold 5 (version 5.1.0). An algorithm for estimating the local false discovery rate (FDR) with standard grouping of proteins was used. To assess the hits of peptides and proteins, FDR = 0.05 was chosen for both. The samples annotated in the Swiss-Prot database were marked as preferred.

Differential gene expression was determined using a log_2_ (fold change) > 0.5 or <−0.5 and false discovery rate < 0.05. PCA was performed on scaled and centered protein counts within the R environment (version 4.4.1).

The interactions between identified differentially secreted proteins were analyzed using the STRING-db online service v. 12.0.

### 4.9. Statistical Analysis

Statistical analysis was performed using GraphPad Prism version 8.03 (GraphPad Software Inc., Boston, MA, USA). Due to the non-normal distribution of the data, the Mann–Whitney U-test was used for comparison. Differences were considered significant at *p* < 0.05.

## 5. Conclusions

AL causes crucial lasting changes in MSCs at the transcriptome, proteome and secretome levels. Those changes affect all the main functions of MSCs: proliferation, differentiation, hematopoietic niche formation and hematopoiesis regulation. Oftentimes, the noted differences are multidirectional, implying dysregulation of MSC metabolism. Upon achieving remission, the studied MSC parameters did not return to the healthy donors’ level. AML-MSCs and ALL-MSCs have both common and individual differences from D-MSCs, suggesting the diverging influence of malignant cells of the two origins, as well as different treatment. With further research, it might be possible to find effective approaches to restoring the stromal microenvironment of AL patients.

## Figures and Tables

**Table 1:** Proteins with differential secretion by MSCs of healthy donors and patients at the onset of AML.

Protein Name	Gene	Average Protein Abundance			*p*-Value, D-MSCs vs. AML-MSCs at the Onset
		Mean, D-MSCs	Mean, AML-MSCs at the Onset	Mean, AML-MSCs in Remission	
Upregulated in patients’ MSCs compared to donors’					
Annexin A1	*ANXA1*	12	19	18.54	0.002714
Annexin A5	*ANXA5*	25	35	34.48	0.008071
Annexin 2	*ANXA2*	29	45	36.16	0.003149
Cadherin-13		4	6	4.98	0.008728
Glucosidase 2 subunit beta	*PRKCSH*	6	11	7.42	0.001226
Insulin-like growth factor-binding protein	*IGFBP5*	3	10	3.96	0.004109
Insulin-like growth factor-binding protein 7	*IGFBP7*	42	64	57.95	0.002819
Lactotransferrin		15	24	20.81	0.023738
LIM and SH3 domain protein 1	*LASP1*	4	6	5.54	0.025224
L-lactate dehydrogenase A chain	*LDHA*	22	32	29.40	0.003608
L-lactate dehydrogenase	*LDHB*	7	11	9.87	0.011045
Mesenchymal stromal cell- and fibroblast-expressing Linx paralogue	*ISLR*	8	10	13.17	0.01979
Metalloproteinase inhibitor 2	*TIMP2*	17	10	10.46	4.33 × 10^−5^
Osteonectin	*SPARC*	106	155	154.23	0.013828
Prosaposin (Variant Gaucher disease and variant metachromatic leukodystrophy) variant (Fragment)		13	25	15.05	0.003819
T cell receptor alpha joining 56 (Fragment)	*TRAJ56*	5	13	13.27	0.016566
Vimentin	*VIM*	110	218	213.61	0.000129
Downregulated in patients’ MSCs compared to donors’					
Adipocyte enhancer-binding protein 1	*AEBP1*	8	4	4.58	0.008281
Annexin (Fragment)	*ANXA6*	8	2	2.97	1.94 × 10^−5^
Anti-RhD monoclonal T125 kappa light chain		4	0	0.10	2.94 × 10^−6^
Antithrombin-III	*ATIII-R2*	5	2	2.07	0.005586
Asparaginyl endopeptidase		6	4	2.08	0.005286
ATP synthase subunit beta, mitochondrial	*ATP5F1B*	3	1	0.71	9.27 × 10^−5^
Beta-2-microglobulin	*B2M*	14	9	10.29	0.004794
CD109 antigen	*CD109*	6	2	1.66	1.35 × 10^−5^
CD166 antigen	*ALCAM*	7	1	1.12	1.97 × 10^−6^
Chondroitin sulfate proteoglycan 4	*CSPG4*	14	3	3.05	0.000144
Complement C3	*C3*	17	7	5.44	0.001488
Complement C4-A	*C4A*	5	2	1.87	0.026311
Complement factor H	*CFH*	22	10	10.26	0.00069
Glucose-6-phosphate isomerase	*GPI*	9	7	6.33	0.021513
Growth arrest-specific protein 6	*GAS6*	12	3	3.56	1.36 × 10^−5^
Insulin-like growth factor-binding protein 2	*IGFBP2*	17	6	5.50	1.32 × 10^−5^
Insulin-like growth factor-binding protein 6	*IGFBP6*	13	7	8.80	0.001175
Integrin beta	*ITGB1*	3	1	0.98	0.000335
LAMA4 protein	*LAMA4*	40	21	23.76	0.000105
Latent-transforming growth factor beta-binding protein 1	*LTBP1*	17	4	4.14	7.18 × 10^−6^
Latent-transforming growth factor beta-binding protein 2	*LTBP2*	63	35	36.54	1.54 × 10^−5^
Macrophage colony-stimulating factor	*CSF1*	4	1	0.79	4.86 × 10^−5^
Matrix metallopeptidase 15 (Membrane-inserted)	*MMP15*	18	0	0.08	0.000378
Peroxidasin homolog	*PXDN*	24	12	14.90	0.000553
Semaphorin-7A	*SEMA7A*	14	6	6.04	3.33 × 10^−6^
Stem cell growth factor; lymphocyte secreted C-type lectin	*CLEC11A*	5	2	3.56	0.005971
Thrombospondin-1	*THBS1*	197	151	171.25	0.009419
Thrombospondin-2	*THBS2*	47	29	25.16	0.001546
Transforming growth factor beta	*TGFB1*	3	0	0.22	1.05 × 10^−5^
Vascular cell adhesion protein 1	*VCAM1*	11	4	4.49	0.001049

**Table 2:** Proteins with differential secretion by MSCs of healthy donors and ALL patients.

Protein Name	Gene	Average Protein Abundance			*p*-Value, D-MSCs vs. ALL-MSCs at the Onset
		Mean, D-MSCs	Mean, ALL-MSCs at the Onset	Mean, ALL-MSCs in Remission	
Upregulated in patients’ MSCs compared to donors’					
Biglycan (Fragment)	*BGN*	0.3	4.2	3.4	0.0023
Isoform 3 of Collagen alpha-1(XVIII) chain	*COL18A1*	0.1	1.4	1.7	0.0007
Actin, cytoplasmic 1 (Fragment)	*ACTB*	0.6	4.0	5.2	0.0132
Collagen alpha-1(III) chain	*COL3A1*	44.8	77.6	146.8	0.0474
Isoform 1 of Fibronectin	*FN1*	5.9	35.3	33.4	0.0473
Protein disulfide-isomerase A3 (Fragment)	*PDIA3*	0.6	2.2	2.6	0.0468
Stathmin	*STMN1*	1.5	7.7	5.1	0.0264
Small ubiquitin-related modifier 3	*SUMO3*	0.6	1.4	1.7	0.0468
Testis-specific serine/threonine-protein kinase 4	*TSSK4*	1.6	9.8	11.8	0.0135
Vascular cell adhesion protein 1	*VCAM1*	1.6	1.8	1.7	0.0135
Protein YIPF3	*YIPF3*	0.4	2.6	1.7	0.0157
Downregulated in patients’ MSCs compared to donors’					
Cathepsin B	*CTSB*	21.5	14.7	8.3	0.0316
Calmodulin 1 (Phosphorylase kinase, delta), isoform CRA_a	*HEL-S-72*	11.8	3.1	2.3	0.0473
Heparan sulfate proteoglycan 2 (Perlecan), isoform CRA_b	*HSPG2*	66.4	16.3	12.2	0.0474
Lysyl oxidase homolog 2 1	*LOXL2*	34.2	3.9	9.0	0.0080
Ribonuclease inhibitor	*RNH1*	9.3	2.4	0.9	0.0316
Thrombospondin-1	*THBS1*	197.4	93.1	113.4	0.0080

**Table 3:** Ratio of average protein abundance in secretome to average protein abundance in proteome (Sec/Pro) for AML-MSCs and D-MSCs.

Compared Groups	Genes
Sec/Pro is higher in AML-MSCs at the onset than in D-MSCs	*FLNA*, *PDAP1*, *KCTD12*, *PSMB4*, *PLBD2*, *LMNA*, *HEXB*, *PDIA3*, *SERPINB2*, *PNP*, *MYL6*, *TAGLN*, *UBE2L3*, *CTSD*, *GDI1*, *GLRX*, *CTSZ*, *SDF4*, *SERPINE2*, *COL5A2*, *CTSB*
Sec/Pro is lower in AML-MSCs at the onset than in D-MSCs	*ANXA6*, *PLEC*, *LRP1*, *SEPTIN7*, *PLIN3*, *HNRNPK*, *CAP1*, *STIP1*, *AHNAK*, *DPYSL2*, *CLIC1*, *TRA1*, *HSP90B1*, *PRDX5*, *MRC2*, *ACTG1*, *TUBB*, *RPS14*, *RPS12*, *PGLS*, *LGALS3*
Sec/Pro is higher in AML-MSCs in remission than in D-MSCs	*ZYX*, *MMP14*, *ACTB*, *VCP*, *HEL-S-70*, *NPM1*, *COL6A3*, *VIM*, *HEL113*, *ENO1*, *HEL-S-17*, *ANXA5*, *HEL-S-7*, *GDI1*, *TAGLN*, *PXDN*, *PPIA*, *HEL-S-69p*, *HTRA1*, *SERPINE2*, *FN1*
Sec/Pro is lower in AML-MSCs in remission than in D-MSCs	*HSPD1*, *PSMD1*, *ANXA6*, *EEF1G*, *MAP1B*, *FERMT2*, *PLEKHC1*, *SPTAN1*, *UBA1*, *UBE1*, *DPYSL2*, *AHNAK*, *FLNB*, *MAP1A*, *TLN1*, *CNDP2*, *SUB1*, *PC4*, *ARCN1*, *HNRNPK*, *PSMB2*, *ACLY*, *PRDX5*, *OLA1*, *DSTN*, *HEL32*, *EEF2*, *LMNA*, *KCTD12*
Sec/Pro is higher in AML-MSCs at the onset than in remission	*HTRA1*, *S100A11*, *SERPINB2*, *PPIA*, *PARK7*, *PFN1*, *CTSZ*, *COL5A2*, *FBLN1*
Sec/Pro is lower in AML-MSCs at the onset than in remission	*ANXA6*, *ARCN1*, *MAP1A*, *TLN1*, *MYH9*, *HARS1*, *PAFAH1B2*, *GAPDH*, *PEPD*, *EEF2*, *PLOD1*, *TUBB*, *RPS14*, *RNH1*, *FTL*

**Table 4:** Characteristics of patients and donors.

			Age, Years (Median)	Gender, Male/Female
Donors		Gene expression (n = 188)	10–61 (32)	98/90
		Secretome (n = 22)	14–62 (33)	15/7
		Proteome (n = 6)	14–34 (26.5)	5/1
Acute Leukemia	Lymphoblastic	Gene expression (n = 14)	18–55 (27)	5/9
		Secretome (n = 2)	18, 19	1/1
		Proteome (n = 5)	23–55 (40)	4/1
	Myeloid	Gene expression (n = 22)	22–64 (47)	8/14
		Secretome (n = 17)	22–64 (42)	6/11
		Proteome (n = 19)	19–64 (36)	6/13

## Data Availability

The data presented in this study are available from the corresponding author on reasonable request.

## Supplementary Materials

The following supporting information can be downloaded at: https://www.mdpi.com/article/10.3390/ijms252413285/s1.
