# Supplementary figures and images for: Damage of the Bone Marrow Stromal Precursors in Patients with Acute Leukemia at the Onset of the Disease and During Treatment
Source: Int J Mol Sci. 2024 Dec 11;25(24):13285. doi: 10.3390/ijms252413285 (PMC11677965; doi:10.3390/ijms252413285)

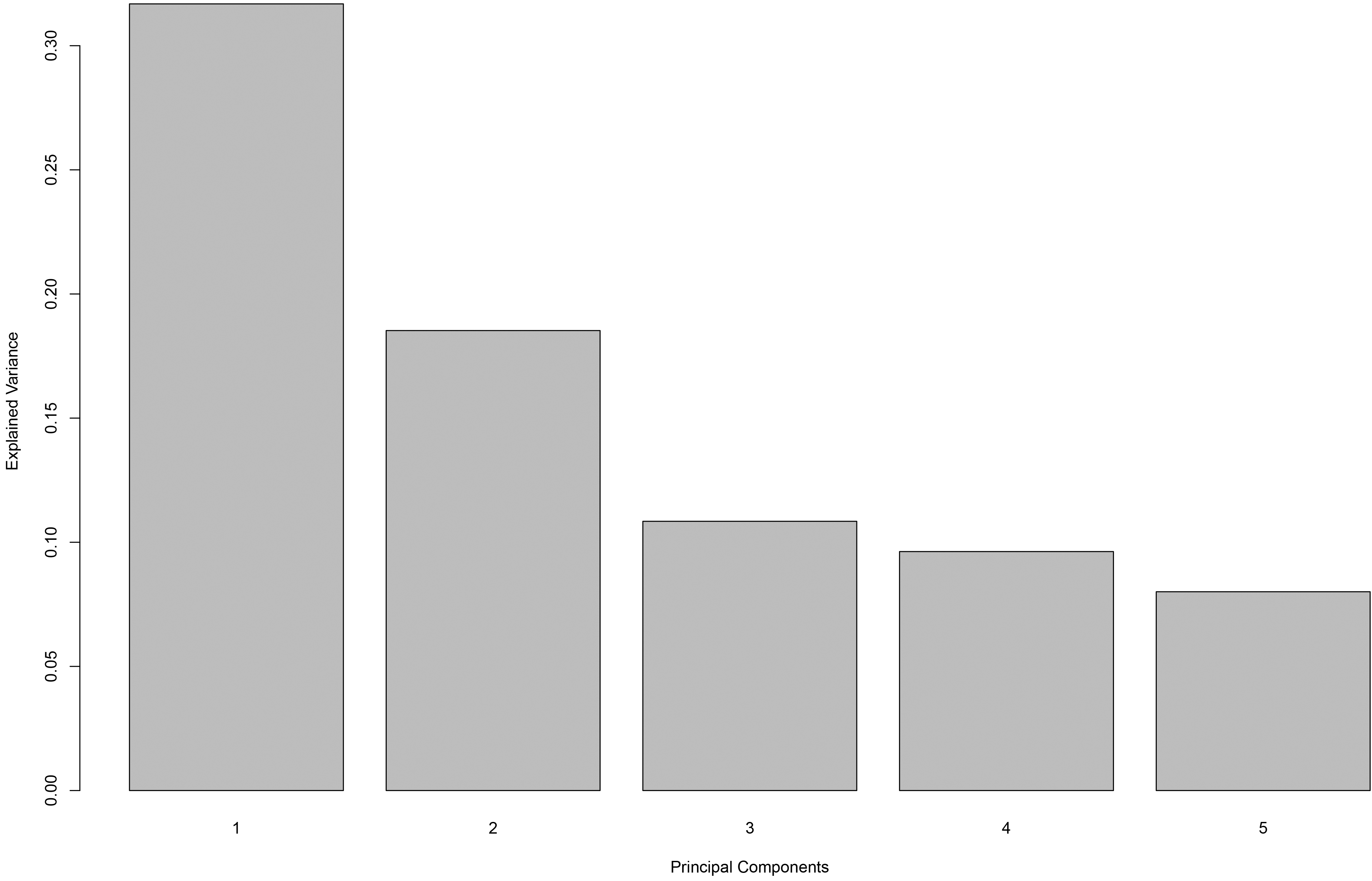

Supplement: Supplementary file 1 [file ijms-25-13285-s001.zip › Suplementary materials/Figure S1 Secretome_Scaffold_PCA-1.jpg]
